# Investigation of a combination therapy approach for the treatment of melioidosis

**DOI:** 10.3389/fmicb.2022.934312

**Published:** 2022-08-16

**Authors:** Kay B. Barnes, Mark I. Richards, Gary Burgess, Stuart J. Armstrong, Christine Bentley, Thomas C. Maishman, Thomas R. Laws, Michelle Nelson, Sarah V. Harding

**Affiliations:** ^1^Defence Science and Technology Laboratory, Porton Down, Salisbury, United Kingdom; ^2^MerLion Pharmaceuticals, Berlin, Germany; ^3^Department of Respiratory Sciences, University of Leicester, Leicester, United Kingdom

**Keywords:** *Burkholderia pseudomallei*, therapy, melioidosis, antibiotics, mice

## Abstract

The efficacy of finafloxacin as a component of a layered defense treatment regimen was determined *in vitro* and *in vivo* against an infection with *Burkholderia pseudomallei*. Doxycycline was down-selected from a panel of antibiotics evaluated *in vitro* and used in combination with finafloxacin in a Balb/c mouse model of inhalational melioidosis. When treatment was initiated at 24 h post-infection with *B. pseudomallei*, there were no differences in the level of protection offered by finafloxacin or doxycycline (as monotherapies) when compared to the combination therapy. There was evidence for improved bacterial control in the groups treated with finafloxacin (as monotherapies or in combination with doxycycline) when compared to mice treated with doxycycline. Survival comparisons of finafloxacin and doxycycline (as monotherapies) or in combination initiated at 36 h post-infection indicated that finafloxacin was superior to doxycycline. Doxycycline was also unable to control the levels of bacteria within tissues to the extent that doxycycline and finafloxacin used in combination or finafloxacin (as a sole therapy) could. In summary, finafloxacin is a promising therapy for use in the event of exposure to *B. pseudomallei.*

## Introduction

*Burkholderia pseudomallei* is the causative agent of the tropical disease melioidosis. It has been estimated that there are 165,000 melioidosis cases per year worldwide, of which 89,000 (54%) patients die (Limmathurotsakul et al., 2016). The treatment options for melioidosis are limited due to the intrinsic resistance of this pathogen to many antibiotics and the risk of relapse of infection at a later date. It has been well-described how *B. pseudomallei* can persist intracellularly for extended periods of time, which also complicates treatment and increases the risk of relapse. Retrospective studies have demonstrated that 4.7 and 9.7% of patients relapsed with the disease in Australia and Thailand, respectively (Limmathurotsakul et al., 2006; Sullivan et al., 2020).

Currently, melioidosis treatment guidelines recommend intravenous administration of ceftazidime or meropenem for a minimum of 14 days initially, followed by 3–6 months of oral dual combination therapy typically comprising co-trimoxazole or co-amoxiclav. However, *B. pseudomallei* resistance to each of these antibiotics has been reported. A recent report evaluated the antibiotic profile of 164 strains of *B. pseudomallei*, and showed that 12.8% were resistant to ceftazidime and 9.8% were resistant to co-trimoxazole (Rao et al., 2019). Meropenem resistance has also been reported, in addition to six *B. pseudomallei* strains in Malaysia that were found to be multi-drug (meropenem, imipenem, and ceftazidime) resistant by disk diffusion assays (Khosravi et al., 2014; Madden et al., 2021; Schnetterle et al., 2021). Furthermore, the rate of adverse events associated with current melioidosis treatment regimens is as high as 30.0%, particularly in response to co-trimoxazole use (Sullivan et al., 2019). In patients with glucose-6-phosphate dehydrogenase deficiency, co-trimoxazole treatment also poses a risk of hemolytic anemia, in addition to causing neurological and kidney problems (Chan, 1997; Ho and Juurlink, 2011).

Melioidosis commonly presents as a respiratory infection. Infection of the airway surface liquid and the alveolar subphase fluid of the lung and the inflammation that results from infection can result in a reduction of pH (Punnia-Moorthy, 1987; De Backer, 2003; Ng et al., 2004). Treating bacterial infections in these acidic environments can be less effective than at neutral pH, particularly observed for the fluoroquinolones and folic acid synthesis inhibitors (Smith et al., 1988; Akova et al., 1999; Yang et al., 2014).

Finafloxacin is a fifth-generation fluoroquinolone that is currently being developed as a treatment option for urinary tract infections and pyelonephritis (Wagenlehner et al., 2018). It has superior activity against both Gram-negative and Gram-positive organisms in conditions of low pH, typical of infected body sites (Higgins et al., 2010; Lemaire et al., 2011; Stubbings et al., 2011). The activity of other antibiotics has been shown to be less active when compared to finafloxacin in clinical trials (Vente et al., 2018; Wagenlehner et al., 2018).

Previous work performed at Dstl has demonstrated broad spectrum *in vitro* activity of finafloxacin against *Francisella tularensis, Yersinia pestis, Coxiella burnetii, Bacillus anthracis, Burkholderia pseudomallei*, and *Burkholderia mallei* (Barnes et al., 2019a; Peyrusson et al., 2021*). In vivo* efficacy has also been demonstrated against *F. tularensis, Y. pestis, C. burnetii*, and *B. pseudomallei* (Barnes et al., 2017, 2019b,^2021^; Hartley et al., 2021). It has been suggested that this is due to the rapid influx of finafloxacin into cells, the accumulation to high levels within the cell, and a slow efflux rate (Chalhoub et al., 2019).

Combination therapy approaches are routinely used to treat a range of diseases, combining different antibiotics, antibiotics and immunomodulators, and antibiotics and antibodies. The use of the fluoroquinolones has been previously investigated in combination with other antibiotics, such as rifampicin (for the treatment of *Mycobacterium ulcerans*), beta-lactams (for the treatment of severely ill patients with bacteremia caused by Gram-negative bacilli), and doxycycline (for the treatment of infections with resistant *Mycoplasma genitalium)* (Al-Hasan et al., 2009; O’Brien et al., 2012; Durukan et al., 2020). In all cases, the combination therapy was more successful at treating the infections. In addition, *in vitro* synergy has been previously demonstrated with finafloxacin combined with amikacin and meropenem against *Escherichia coli* and *Pseudomonas aeruginosa* (Goh et al., 2011).

Although modest *in vivo* efficacy of finafloxacin monotherapy against an infection with *B. pseudomallei* has been previously demonstrated, its utility as a component in combination therapy is yet to be explored. Therefore, the aim of this study was to investigate the *in vitro* activity and *in vivo* efficacy of finafloxacin in combination with doxycycline. Using an inhalational model of melioidosis, the survival and eradication of colonizing bacteria following a 14-day monotherapy or combination therapy were compared.

## Materials and methods

### Bacteria

#### *In vitro* assays

*Burkholderia pseudomallei* strain K96243 (obtained from the UK Health Security Agency) was prepared by adding 10 μL of a frozen bacterial stock to 10 mL of cation-adjusted Mueller-Hinton broth (CAMHB) and incubating at 37°C with shaking at 180 rpm for 24 h.

#### *In vivo* study

*Burkholderia pseudomallei* strain K96243 was streaked onto a Luria agar (L-agar) plate and incubated at 37°C for 24 h. The following day, a loopful of bacteria was re-suspended into 10 ml of phosphate-buffered saline (PBS) and adjusted to an OD_590_ of 0.36. One milliliter of this was inoculated into 100 ml of Luria-broth (L-broth). This was incubated at 37°C with shaking at 180 rpm for 16 h and adjusted to approximately 1 × 10^8^ CFU/mL. A 1:50 dilution was then performed in sterile PBS, and this dilution was used for the efficacy study. All bacteriological procedures were carried out in Class III microbiological safety cabinet or Class III half-suit rigid isolator within an Advisory Committee on Dangerous Pathogens (ACDP) Containment Level 3 laboratory.

### Animals

Animal studies were carried out in accordance with the UK Animals (Scientific Procedures) Act 1986, the codes of practice for the Housing and Care of Animals used in Scientific Procedures 1989, and an ACURO Appendix. Female BALB/c mice (Charles River Laboratories, United Kingdom) aged 8–10 weeks were randomized into cages with five animals in each cage and stored within a rack in an ACDP Containment Level 2 laboratory (pharmacokinetics study) or in a rack within a Class III half-suit rigid isolator in an ACDP Containment Level 3 laboratory (efficacy study). Mice had free access to water and a rodent diet (Harlan Teklad, United Kingdom) and underwent a 5-day acclimatization period before any procedures were performed.

### Antibiotics

Finafloxacin HCl salt was supplied by MerLion Pharmaceuticals Ltd. Doxycycline, meropenem, and co-trimoxazole were purchased from Sigma. For the *in vitro* assays, working concentrations of 10 mg/mL of finafloxacin were prepared by adding 118 mg of finafloxacin salt (containing 100 mg of active antibiotic) to 9 mL of sterile water and 1 mL of 1M sodium hydroxide. The co-trimoxazole working solution was prepared by adding 167 mg of sulfamethoxazole to 9 mL of sterile water and 1 mL of 1M sodium hydroxide. 33 mg of trimethoprim was added to 10 mL of sterile water and 30 μL of acetic acid. The solutions were mixed, and the resulting co-trimoxazole was used at a ratio of 5:1 (sulfamethoxazole:trimethoprim). Meropenem and doxycycline were prepared by making a 10 mg/ml solution by dissolving in distilled water. Bacteria grown in the equivalent concentration of sodium hydroxide or acetic acid, used to prepare the antibiotics, were included as a control.

For the *in vivo* study, a 15 mg/mL solution of finafloxacin was prepared by adding 2.1 mL of 0.01M Tris buffer to 44 mg of finafloxacin powder (containing 37.5 mg of active ingredient). 200 μL of 1 M sodium hydroxide was added to dissolve the antibiotic followed by 200 μL of 0.01 M hydrochloric acid. The pH of the resulting solution was 8. Doxycycline monohydrate (referred to as doxycycline for the remainder of the manuscript) was purchased from Pfizer Limited (UK). One doxycycline tablet was reconstituted in 1 mL of distilled water.

### Checkerboard assays

Overnight cultures of *B. pseudomallei* grown in L-broth were diluted to 0.25 at OD_600_, and 100 μl was added to 10 mL of CAMHB. Assays were performed at pH 5 and pH 7 using finafloxacin in combination with meropenem, co-trimoxazole, or doxycycline (Garcia, 2007). The level of antibiotic-induced inhibition was determined with combinations scored based on the sum of the fractional inhibitory concentration (ΣFIC).

The ΣFICs were calculated as follows: ΣFIC = FIC A + FIC B, where FIC A is the MIC of drug A in the combination/MIC of drug A alone, and FIC B is the MIC of drug B in the combination/MIC of drug B alone (Garcia, 2007). A combination was considered synergistic when the ΣFIC was ≤0.5, indifferent when the ΣFIC was >0.5 to <4, and antagonistic when the ΣFIC was ≥4.

### Time-kill assays

Time-kill assays of the antibiotic combinations were performed at multiples of the MIC (1 X and 2 X) (Wayne, 1999; Garcia, 2007). Antibiotic solutions of finafloxacin, co-trimoxazole, or doxycycline were prepared in 10 mL of CAMBH adjusted to pH 5 or pH 7.

Broths were inoculated with *B. pseudomallei* at a concentration of approximately 5 × 10^5^ CFU/mL. Control samples contained bacteria cultured in the absence of antibiotics. All broths were incubated with shaking at 180 rpm at 37°C. Samples were taken at 0, 2, 4, 6, and 24 h, a 10-fold serial dilution was performed in PBS, plated onto L-agar, and incubated at 37°C. Colonies were enumerated following incubation for 48 h. Synergy was defined as a ≥2 log reduction from the most active antibiotic used as monotherapy over a 24-h period. Antagonism was defined as a ≥2 log increase from the most active antibiotic used as monotherapy over a 24-h period (Garcia, 2007).

Time-kill assays were performed, using finafloxacin combined with doxycycline or co-trimoxazole at 1 × MIC and 2 × MIC. The 1 × MIC was 1 μg/ml (at pH 5) and 4 μg/ml (at pH 7) for finafloxacin, 1 μg/ml (pH 5) and 0.5 μg/ml (pH 7) for doxycycline, and 16 μg/ml (pH 5 and pH 7) for co-trimoxazole. These assays were used to determine the killing capacity of the antibiotics, when evaluated as a monotherapy or in combination. Synergy in this assay was defined as a ≥2 log10 reduction in CFU/mL from the most active monotherapy (Garcia, 2007).

### Pharmacokinetic study

To determine the *in vivo* pharmacokinetics, a single dose of doxycycline (150 mg/kg) was delivered in a 30 μL volume by the oral route (*via* pipette tip) in Balb/c mice. Alternatively, a 30 μL dose of doxycycline (150 mg/kg) was delivered with finafloxacin (23.1 mg/kg) in a 31 μL volume as the active salt by the oral route. Blood was collected under terminal anesthesia into lithium heparin tubes from five mice per time point at 0.5, 1, 2, 4, 6, 8, 12, and 24 h post-dosing. Plasma was separated from the whole blood and stored at −80°C prior to analysis by Swiss BioQuant (Reinach, Switzerland). The mean antibiotic concentration–time profile was generated and analyzed using Phoenix WinNonlin v 8.0 (Certara Inc.) to calculate various parameters, including the maximum drug concentration (C_max_), the time taken to reach C_max_ (T_max_), the area under the curve (AUC), the clearance rate (CL), the volume of distribution (V), and the terminal half-life (T½).

### *In vivo* efficacy study

Aerosol inhalation was used to establish the *B. pseudomallei* infection in mice. Animals were restrained within a nose-only exposure tube and placed within an exposure chamber, which was connected to a Collison three-jet nebulizer and spray tube in an AeroMP apparatus Biaera Technologies (Hagerstown, MD, United States). Fifteen milliliters of bacteria (range of 1.44 × 10^5^ CFU/mL–4.16 × 10^5^ CFU/mL) was placed into the nebulizer, and the mice were exposed for 10 min to a dynamic aerosol conditioned in an Aero MP apparatus. The aerosol stream was maintained at 70 ± 2% relative humidity and 22°C. The concentration of *B. pseudomallei* in the aerosol was determined by recovering samples from the exposure chamber using an All Glass Impinger operating at 12 L/min, containing 10 mL of sterile PBS. Impinger samples were plated onto L-agar for bacterial enumeration, and the retained dose of bacteria that mice received in each run was calculated by applying the Guyton formula (Guyton, 1947). It was assumed that each mouse retained 40% of the organisms that were inhaled (Harper and Morton, 1962).

Treatment was initiated at 24 or 36 h post-infection and continued for 14 days. Groups of 10 mice were administered finafloxacin (23.1 mg/kg), doxycycline (100 mg/kg), both antibiotics in combination, or the vehicle control (Tris buffer) by the oral route (*via* pipette tip) every 8 h. Mice were weighed daily and observed a minimum of twice daily for clinical signs of disease for 42 or 43 days when the experiment was terminated. Clinical scores were determined based on the observed changes in the animals behaviour and condition (including piloerection, hunching, changes to mobility, and respiration). Mice were euthanized *via* a Schedule 1 procedure once they had reached their humane endpoint.

Endpoint analyses were performed at scheduled times throughout the experiment. The first cull was at treatment initiation (24 or 36 h post-infection, prior to treatment initiation), the second following 14 days of treatment, and finally at the end of the study (day 42 or 43 post-infection). A panel of organs (liver, lungs, spleen, kidney, and brain) and blood were harvested, weighed, and processed for bacterial burden. They were homogenized in 1 mL of PBS, a 10-fold serial dilution was performed, and 100 μL aliquots were plated onto L-agar in duplicate. The agar plates were incubated for 2 days at 37°C and enumerated to determine the bacterial load in the organs. The remaining homogenate was diluted 20-fold in L-broth, incubated for a further 5 days at 37°C, then streaked onto L-agar plates, and finally incubated at 37°C for 48 h to confirm the absence of *B. pseudomallei*. Differences in survival, body weight, clinical scores, and bacterial burden were determined.

### Minimum inhibitory concentration

Bacterial isolates recovered from animals that survived the experimental period were assessed for sensitivity to finafloxacin Biomerieux (Hampshire, United Kingdom, 0.002–32 μg/mL) and doxycycline (Biomerieux, Hampshire, United Kingdom 0.016–256 μg/mL) as per the manufacturer’s instructions.

### Statistical analysis

Graphs were prepared using PRISM 8.0 (Graphpad). This software was also used to analyze some datasets. Time kill data were analyzed using a two-way ANOVA with Tukey’s multiple comparison test; however, for some assays where there were several data points below the limit of detection, the variance was not equal and therefore the data were not suitable for this test.

A sequence of log-rank tests using IBM SPSS V27.0 were used to compare the protection offered by the three treatment regimens. Mice were considered censored (either culled or alive) when culled for experimental analysis (i.e., not a lethal endpoint) and if they survived until the end of the study. Stratification was used in some tests using intervention time.

Conventional analysis of variance was not possible when comparing the bacterial load data, as many groups had no variance (due to most organs being clear); therefore, the blood and kidney data were excluded from this analysis. A negative binomial generalized linear model was used to analyze the rest of the data using SPSS. There were four explanatory variables: organ type, specific treatment, treatment initiation time, and time the samples were harvested, and therefore there was a risk of the full factorial model being over-fit. As a consequence, a model building method was used and the Collett method was run (Collett, 2015). A reasonable fit of the model was assumed from a plot of measured and predicted values.

Repeated measures linear models were constructed to analyze the bodyweight data using SPSS. One analysis ran until 15 days post-infection, when the first experimental cull occurred and the other analysis ran to the end of the experiment. Mice that were culled at their humane endpoint (one mouse up to day 15 post-infection and nine mice up to the end of the experiment) were excluded from the analysis, and the models were run on each of the experimental cull days, when significant numbers of mice were removed from the study. The model included time (of cull), treatment initiation time (*24* or 36h), and the treatment regimen (finafloxacin, doxycycline, or the combination). The Greenhouse–Geisser correction was used to account for potential non-sphericity. To dissect the differences between groups, pairwise analyses were performed. Bonferroni’s correction was used to account for multiple tests.

## Results

### Synergy was observed for finafloxacin and meropenem in checkerboard assays

The activity of finafloxacin in combination with meropenem, co-trimoxazole, or doxycycline was determined by checkerboard assay at pH 5 and pH 7. At pH 5, all antibiotic combinations were classed as indifferent (Table 1). At pH 7, synergy was observed at 0.5 μg/ml of finafloxacin and 0.25 μg/ml of meropenem, respectively. All other antibiotic combinations were classed as indifferent.

**TABLE 1:** Checkerboard assays with *B. pseudomallei* detailing the effect of combining finafloxacin with doxycycline, co-trimoxazole, or meropenem at pH 5 or pH 7.

pH 5			
**Finafloxacin (μg/ml)**	**Meropenem (μg/ml)**	**ΣFIC**	**Interpretation**
0.06–0.5	0.12–2	0.6–1.1	Indifferent
	**Co-trimoxazole (μg/ml)**		
0.06–1	1–16	1.1–1.5	Indifferent
	**Doxycycline (μg/ml)**		
0.06–1	0.25–1	0.8–1.3	Indifferent

**pH 7**			

**Finafloxacin (μg/ml)**	**Meropenem (μg/ml)**	**ΣFIC**	**Interpretation**
0.5	0.25	0.5	**Synergistic**
0.06–0.5	0.5–1	0.6–1.1	Indifferent
	**Co-trimoxazole (μg/ml)**		
0.06–1	1–16	1.1–1.5	Indifferent
	**Doxycycline (μg/ml)**		
0.06–0.5	0.25–1	0.8–1.3	Indifferent

### Synergy and antagonism were observed in time-kill assays

**FIGURE 1:**
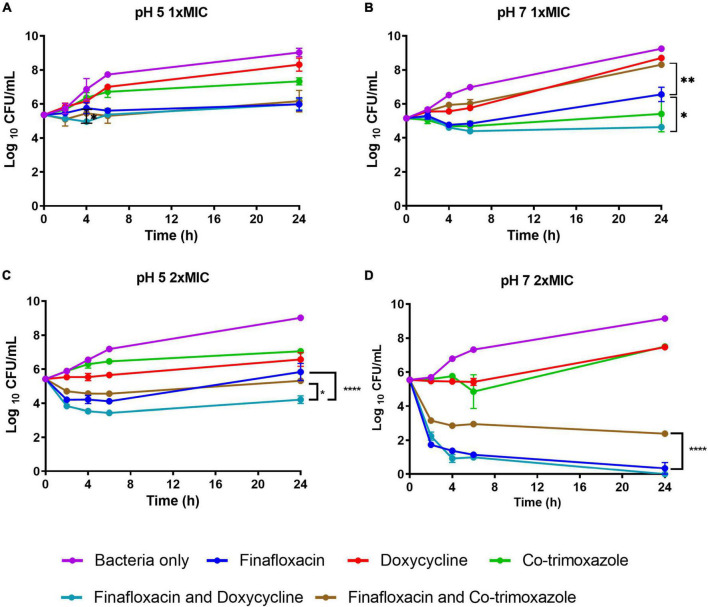
The time-kill assays for *Burkholderia pseudomallei* grown in the presence of antibiotics. These assays were performed in CAMBH adjusted to pH 5 or pH 7. An untreated bacterial control was included. **(A)** pH 5, finafloxacin (1 μg/ml), doxycycline (1 μg/ml), and co-trimoxazole (16 μg/ml). **(B)** pH 7, finafloxacin (4 μg/ml), doxycycline (0.5 μg/ml), and co-trimoxazole (16 μg/ml). **(C)** pH 5, finafloxacin (2 μg/ml), doxycycline (2 μg/ml), and co-trimoxazole (32 μg/ml). **(D)** pH 7, finafloxacin (8 μg/ml), doxycycline (1 μg/ml), and co-trimoxazole (32 μg/ml). The error bars represent the SEM of two biological replicates. *p < 0.05, **p < 0.01, ****p < 0.0001.

At 1 × MIC at pH 5, there was no difference between finafloxacin used as a monotherapy or in combination with doxycycline over 24 h; however, there was a reduction in bacterial concentration at 4 h when the antibiotics were evaluated in combination (*p* < 0.05; Figure 1A). There were no other differences between finafloxacin used as a monotherapy or in combination (Figure 1A). At pH 7, the combination of finafloxacin and doxycycline resulted in a reduction in bacterial concentration when compared to finafloxacin alone (*p* < 0.05) over 24 h, indicating synergy (Figure 1B). There was also a difference between finafloxacin used as a monotherapy and in combination with co-trimoxazole. The combination appeared to be less effective than finafloxacin alone (*p* < 0.01; Figure 1B).

At pH 5, the 2 × MIC combination of finafloxacin with doxycycline was shown to be synergistic (*p* < 0.0001; Figure 1C) and more effective than the finafloxacin/co-trimoxazole combination (*p* < 0.05). At pH 7, the combination of finafloxacin and co-trimoxazole was shown to be antagonistic (*p* < 0.0001; Figure 1D).

The killing profile over time between the antibiotics used at 1 × MIC and 2 × MIC at pH 5 was very similar, except for the finafloxacin/co-trimoxazole combination, which was more active at the higher antibiotic concentrations used and was similar to the profile demonstrated when 1 × MIC was incubated at pH 7 (Figures 1A–C). There was an improvement in the killing ability of finafloxacin combined with co-trimoxazole at 2 × MIC and pH 7 compared to when utilized at 1 × MIC. The use of finafloxacin as a monotherapy or combined with doxycycline at 2 × MIC and pH 7 resulted in approximately a 5-log reduction in bacterial concentration over 24 h, with approximately a 4-log reduction demonstrated within 4 h (Figures 1B,D).

### Co-administration of doxycycline with finafloxacin does not significantly affect the pharmacokinetic profile

The pharmacokinetic (PK) profile of doxycycline delivered in combination with finafloxacin was determined in mice. The combined administration of doxycycline with finafloxacin did not significantly change the PK disposition of doxycycline or finafloxacin (Table 2). A reduction in Cmax was observed; however, the dose of doxycycline used achieved a similar AUC to that reported previously (in Balb/c and A/J mice) (Dstl, unpublished data). The CL for doxycycline in Balb/C mice (8.2 mL/h/kg) appears to be lower than for humans (25–67 mL/h/kg, data normalized to a nominal 70 kg person). The half-life of doxycycline also appears to be reduced when the combination is delivered; however, the PK parameter linked to tetracyclines (e.g., doxycycline) is the AUC_0–24 h_/MIC ratio (Craig, 1998). As the murine equivalent doses at the higher end of the range for AUC for doxycycline in humans (41–123 μg h/mL) are potentially toxic (Agwuh and MacGowan, 2006) (Pfizer Safety Data Sheet: Doxycycline monohydrate for oral suspension), a dose of 100 mg/kg of doxycycline delivered every 8 h was selected to achieve the lower AUC of 41 h⋅mg/L.

**TABLE 2:** The non-compartmental PK parameters determined for doxycycline and finafloxacin in the plasma of Balb/C mice.

Parameter		Doxycycline	Doxycycline (and Finafloxacin)	(Doxycycline and) Finafloxacin	Finafloxacin (SIMULATED)
Dose	mg/kg	150	150	23.1	23.1
T½	h	5.2	2.1	1.7	1.2^‡^
C_max_	ng/mL	3/,220 ± 255	3,634 ± 231	1,934 ± 274	4,229^†^
T_max_	h	0.5	1	0.5	0.1^†^
AUC	h⋅mg/L	17.6 ± 1.5	20.2 ± 1.5	8.2 ± 1.0	8.0^†^
AUC_0–∞_	h⋅mg/L	18.2	20.4	8.6	ND
CL	L/h/kg	8.2	7.3	2.7	2.9^‡^
V	L/Kg	61.5	21.9	6.6	5.0^‡^

The underlined antibiotics are the parameters for that antibiotic.

^†^The data for finafloxacin delivered alone are simulated using a one-compartment model, and parameterized with the data generated from a PK study where 37.5 mg/kg of finafloxacin was delivered.

^‡^Measured parameters determined by a one-compartment model to fit to PK data generated in mice following administration of 37.5 mg/kg of finafloxacin. ND, not determined.

The dose of finafloxacin used in this PK study (23.1 mg/kg) was predicted by modeling data previously generated for a dose of 37.5 mg/kg (Barnes et al., 2017) (presented as “simulated” data in Table 2). In combination with doxycycline, the empirical data from this dose closely matched the predicted PK parameters. In order to achieve the target AUC, which is equivalent to a human administered daily oral dose of 800 mg (26.1 mg⋅h/L) (Patel et al., 2011), 23.1 mg/kg of finafloxacin was delivered every 8 h.

### Rapid dissemination and establishment of a *Burkholderia pseudomallei* infection are observed following inhalational infection

Following aerosol exposure, the mean retained dose of *B. pseudomallei* was 106 CFU (range 81–156 CFU). At 24 h post-infection, some mice displayed minor clinical signs of disease (piloerection), and at 36 h post-infection, mice displayed mild clinical signs (piloerection and hunched posture) and rapid weight loss. No animals succumbed to the disease before the treatment was initiated. The bacterial challenge was lethal, and all control animals (treated with the vehicle) succumbed to infection by day 4 post-infection.

### At treatment initiation, *Burkholderia pseudomallei* is systemic

**FIGURE 2:**
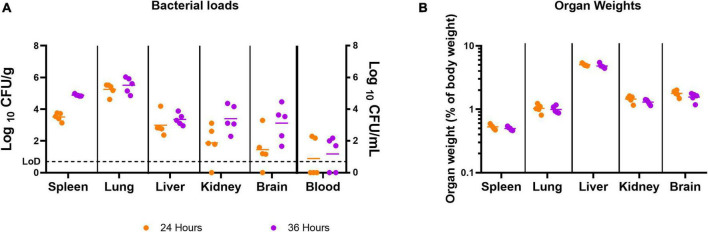
The concentration of *B. pseudomallei* and the weight of organs before treatment was initiated. Bacterial counts (CFU/g or CFU/mL) **(A)** and organ weights **(B)** were determined for a panel of organs at 24 or 36 h post-infection. Five mice were challenged and culled at either 24 or 36 h post-infection. LoD, limit of detection.

At 24 h post-infection, bacteria were detected in 100% of the spleens, livers, and lungs, 80% of the kidneys and brains, and 40% of the blood samples (Figure 2A). At 36 h post-infection, all spleens, livers, lungs, kidneys, and brains, and 60% of the blood samples were colonized. There were no marked differences in the organ weights at these time points (Figure 2B).

### Finafloxacin offered improved protection when compared with doxycycline administered at 36 h post-infection

**FIGURE 3:**
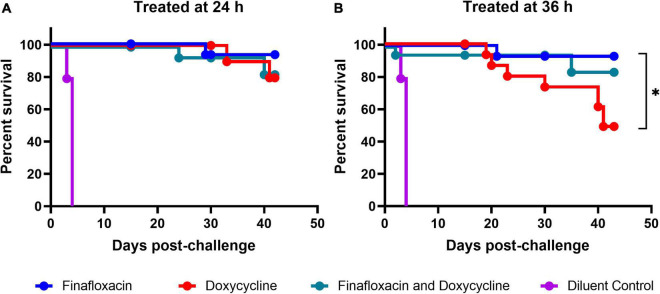
The percentage survival of mice in each treatment group following challenge with aerosolized *B. pseudomallei*. Mice were challenged with a mean retained dose of 106 CFU of *B. pseudomallei* by the inhalational route and received either finafloxacin (23.1 mg/kg), doxycycline monohydrate (100 mg/kg), or both antibiotics in combination, by the oral route every 8 h. Control animals received the vehicle by the oral route. Regimens were initiated at 24 **(A)** or 36 **(B)** h post-infection with mice receiving 14 days of therapy. Survival curves were compared using log-rank (Mantel-Cox) tests. *p < 0.05.

All three regimens offered significant protection in comparison to the vehicle control (*p* < 0.001; Figure 3). When the data were stratified by treatment initiation time, the analysis suggested that there were differences between the three treatments when initiated at 36 h post-infection (*p* = 0.041; Figure 3). No evidence for differences between the three treatment groups was observed when the treatments were initiated at 24 h post-infection (*p* = 0.864; Figure 3A). Pairwise comparisons of the three treatments initiated at 36 h post-infection indicated that there was likely to be a difference between the doxycycline and finafloxacin monotherapies (*p* = 0.026), and the outcome improved for the mice treated with finafloxacin (Figure 3B).

### Clearance of *Burkholderia pseudomallei* infection following finafloxacin treatment

**FIGURE 4:**
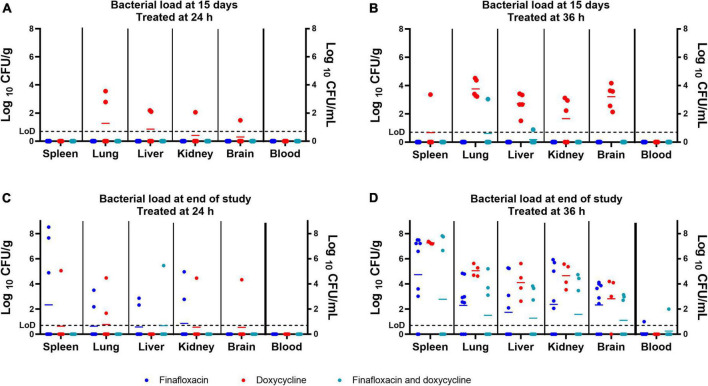
The bacterial load in a panel of organs at different time points throughout the *B. pseudomallei* study. Bacterial counts (CFU/g of tissue or CFU/mL of blood) in organs from mice treated from 24 h post-infection and culled at the end of the treatment regimen (day 15 post-infection) **(A)**, from mice treated from 36 h post-infection and culled at the end of the treatment regimen (day 15 post-infection) **(B)**, from mice treated from 24 h post-infection and culled at the end of the study (day 42 post-infection) **(C)**, and from mice treated from 36 h post-infection and culled at the end of the study (day 43 post-infection) **(D)**. LoD, limit of detection. The colored lines in the columns represent the mean of the group. The data associated with this figure was analyzed in the model used to generate Figure 5.

Following the completion of antibiotic treatment (day 15 post-infection), no bacteria were detected in any of the organs harvested from mice treated with finafloxacin or the combination of finafloxacin and doxycycline when treatment was initiated at 24 h post-infection (Figure 4A). Following treatment with doxycycline, *B. pseudomallei* was recovered from the lung, liver, kidney, and brain of one mouse and the lung, liver, and kidney of a second mouse. There were no detectable bacteria in any of the blood samples.

No bacteria were detected in any of the organs harvested from mice treated with finafloxacin initiated at 36 h post-infection (Figure 4B). *B. pseudomallei* was recovered from the lung and liver of a single mouse treated with the combination of finafloxacin and doxycycline. All mice treated with doxycycline had bacteria in the lungs, liver, kidneys, and brain, and three mice had bacteria in the spleen. No bacteria were detected in any of the blood samples.

### Clearance of systemic *Burkholderia pseudomallei* infection was improved with combination therapy

Animals surviving up to day 42 or 43 post-infection were euthanized to determine the level of clearance within a panel of organs. Bacteria were detected in the spleen of four animals, the lungs of three, and the livers, kidneys, and brain of two, when treatment was initiated at 24 h post-infection (Figure 4C and Table 3). Four of the nine surviving mice treated with finafloxacin had completely cleared the infection (Table 3). *B. pseudomallei* was recovered from the liver, spleen, lung, kidney, and brain of one mouse treated with the combination. Seven of the surviving eight mice treated with the combination had completely cleared the infection (Table 3). Bacteria were detected from one mouse treated with doxycycline in the spleen, lung, liver, kidney, and brain and from the liver and lung of two additional animals. Five of the eight surviving mice treated with doxycycline had completely cleared the infection (Table 3). There were no bacteria detected in the blood.

**TABLE 3:** The number of mice clear from colonizing bacteria at day 42 or 43 post-infection.

Treatment	Initiated at 24 h post-infection	Initiated at 36 h post-infection	Total mice clear
Finafloxacin	4/9	1/9	5/18
Doxycycline	5/8	0/4	5/12
Combination	7/8	4/8	11/16

**FIGURE 5:**
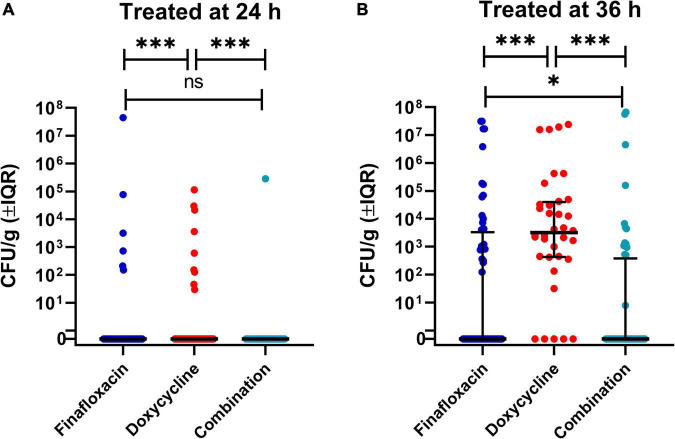
The bacterial load in a panel of organs at different time points throughout the *B. pseudomallei* study. This analysis investigated the concentration of bacteria in tissues at both time points (day 15 and 42/3 post-infection), the different treatments, and treatment initiation time, 24 h **(A)** and 36 h post-infection **(B)**. The significance markers are derived from indicative of Bonferroni’s corrected negative binomial generalized linear model Wald tests. Each dot represents an individual organ. These graphs use a representation of a log10 scale that allows the visualization of zero bacteria (marked 0) and one single bacterium (at 10^0^). The lines are the median values for each group and the error bars are the interquartile range. This is a representation of a key interaction of a statistical model. ^***^p < 0.001, ^**^p < 0.01, *p < 0.05. ns, not significant.

To be determined as clear of colonizing bacteria, all tissues and blood had to be negative by plate count on solid agar and following incubation of the remaining homogenate in liquid media. The bacterial load data associated with this table were included in the statistical models (Figure 5).

Seven of the surviving nine mice treated with finafloxacin initiated at 36 h had detectable bacteria in the spleen and lungs, five mice in the liver, six in the kidney and brain, and one in the blood (Figure 4D and Table 3). One mouse had completely cleared the infection. *B. pseudomallei* was recovered from the spleen, lungs, liver, and kidneys of three mice treated with the combination of the brain of four animals and the blood of one animal. Four out of the eight surviving mice treated with the combination had completely cleared the infection (Figure 4D and Table 3). All four surviving mice treated with doxycycline had bacteria in the spleen, lung, liver, kidney, and brain, and one mouse also had bacteria in the blood. Overall, more mice treated with the combination had cleared the infection at the end of the study compared to the animals treated with finafloxacin or doxycycline (Table 3).

The data with regard to bacterial load from the scheduled cull at day 15 post-infection and at the end of the study were analyzed together (Figure 5). The organ type, treatment initiation time, and time point (of the cull) showed a probable effect on bacterial load (*p* = 0.001), with the exception of the interaction between the time point (of the cull) and treatment initiation time (*p* = 0.317). Pairwise analysis was used to characterize the role of the specific treatments in the model. The bacterial load in the organs of the doxycycline-treated group was different from mice treated with the combination or finafloxacin monotherapy (*p* = 0.001) at day 15 post-infection. At the end of the study period, there were differences in bacterial load between the animals treated with the combination and the finafloxacin or doxycycline monotherapies (*p* = 0.001). When treatment was initiated at 24 h post-infection, the bacterial load in organs harvested from the doxycycline-treated animals was different from the load observed in the combination and the finafloxacin-treated animals (*p* = 0.001). When treatment was initiated at 36 h post-infection, there were differences between the bacterial load in organs harvested from all treatment groups (*p* = 0.001), except for finafloxacin compared to the combination (*p* = 0.050).

There was also evidence for different treatment effects in different organs (*p* = 0.001). Differences between the bacterial load were observed in the brains and spleens harvested from the doxycycline animals and those treated with the combination and finafloxacin monotherapy (*p* = 0.039 and *p* = 0.015), respectively. In the liver, there was a difference in the bacterial load between the finafloxacin- and doxycycline-treated groups (*p* = 0.022).

### Finafloxacin as monotherapy was superior at limiting initial weight loss

**FIGURE 6:**
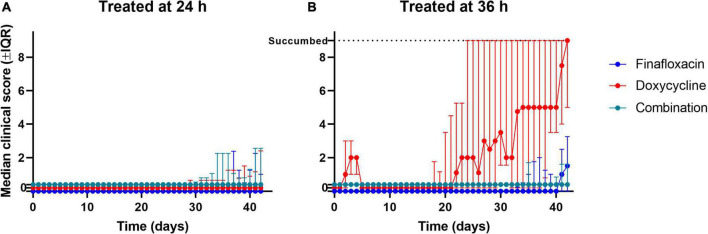
The clinical scores recorded throughout the study. Mice were challenged with a mean retained dose of 106 CFU of *B. pseudomallei* by the inhalational route and received either finafloxacin (23.1 mg/kg), doxycycline monohydrate (100 mg/kg), or both antibiotics in combination, by the oral route every 8 h. Control animals received the vehicle by the oral route. Regimens were initiated at 24 **(A)** or 36 **(B)** h post-infection with mice receiving 14 days of therapy. Clinical scores were recorded a minimum of two times daily. The data are shown as the median value for each group at each time point, and the error is the interquartile range. When mice reached their lethal endpoint, an arbitrary value of 9 (one greater than the maximum) was assigned for all subsequent time points. Scores of zero have been offset between groups to help with visualization.

The body weights were recorded daily, and two analyses were conducted for each of the scheduled cull points (day 15 post-infection and the end of the study) (Figure 6). For the analysis including animals that survived until the end of the study (finafloxacin,*n* = 18; doxycycline,*n* = 12; and combination,*n* = 16), differences were suggested between the treatment initiation times (*p* = 0.009, as interaction with time). However, any effect observed in this stratum of the data may be biased by the fact that the mice that succumbed to the disease were not included in the analysis (finafloxacin,*n* = 2; doxycycline,*n* = 8; and combination,ñ=4).

For the animals that survived until day 15 post-infection (finafloxacin,*n* = 30; doxycycline,*n* = 30; and combination, *n* = 29), there was evidence to suggest differences between the treatment groups (*p* = 0.001, as interaction with time) and between the time of treatment initiation (*p* = 0.001, as interaction with time). There was evidence of a reduction in weight loss for the mice treated with finafloxacin compared to those treated with doxycycline or the combination (both *p* = 0.012as interaction with time). When the animals treated with doxycycline were compared to the animals treated with the combination, there was also a difference (*p* = 0.030, as interaction with time). A single mouse was excluded from this stratum, so the likelihood of bias was minimal.

**FIGURE 7:**
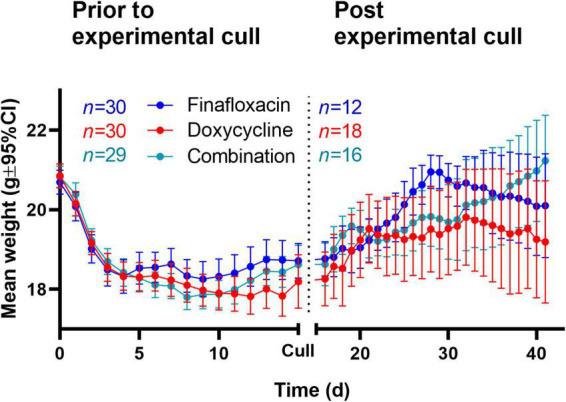
The weight loss observed in mice infected with *B. pseudomallei* and treated with antibiotics. Mice were challenged with a mean retained dose of 106 CFU of *B. pseudomallei* by the inhalational route and received either finafloxacin (23.1 mg/kg), doxycycline monohydrate (100 mg/kg), or both antibiotics in combination, by the oral route every 8 h, initiated at 24 and 36 h post-infection. Control animals received the vehicle by the oral route. Mice were weighed daily. The data show the mean of multiple mice (*n* given before and after the cull at the end of the treatment) and the 95% confidence interval generated by the model. The dotted line represents the scheduled cull point at the end of the treatment regimen. All mice (treated from 36 and 48 h post-infection) with all treatments are included in this figure.

The administration of treatment at different time points post-infection affects the weight profiles independent of the treatment regimen. How the different treatments affected changes in weight appears to be time-specific and complex; however, in general, finafloxacin monotherapy was superior at limiting initial weight loss (Figure 7).

### Treatment with the combination prevented the development of severe clinical scores of infection

The mice that were treated with the three different antibiotic regimens initiated at 24 h post-infection developed mild (low) clinical scores from day 25 post-infection (Figure 6A). At the end of the study period, two mice treated with finafloxacin had a clinical score of 1, while the remaining mice had no clinical score reported.

When treatment was initiated at 36 h post-infection, mice treated with finafloxacin or the combination developed mild (low) clinical scores from day 28 post-infection until the end of the study (Figure 6B). The mice treated with finafloxacin developed further signs from day 35 post-infection onward, which aligns with the bacterial load data at the end of the study, where more of the finafloxacin-treated mice were colonized. Of the nine surviving mice treated with finafloxacin, three had a score of 3 and one had a score of 4 at the end of the study period, while the remaining mice had no clinical score reported. Of the eight surviving mice treated with the combination, two mice had a score of 2 at the end of the study, and again the remaining mice had no clinical score reported.

The mice treated with doxycycline developed clinical signs of infection for the first 8 days post-infection which were resolved; however, following treatment cessation at day 15 post-infection, they again developed signs of infection. At the end of the study, the five surviving mice had an average clinical score of 5. When treatment was delayed until 36 h post-infection, doxycycline failed to prevent clinical signs from developing once treatment had stopped.

### No antibiotic resistance was observed

The MICs were determined for five isolates harvested from the surviving animals treated with the monotherapies or the combination. No evidence of resistance was observed, as the MICs were equivalent to or less than that those observed for wild-type K96243, that is, 4 and 1 μg/ml for finafloxacin and doxycycline, respectively.

## Discussion

The treatment of infections caused by *B. pseudomallei* is difficult, due to the resistance of the bacterium to many antibiotics, the length of the current treatment regime, and the potential for relapse of the disease. The identification of novel treatments and alternative treatment strategies is warranted. A combination therapy approach was investigated, initially combining the fluoroquinolone finafloxacin with the antibiotics of different classes, such as meropenem (a carbapenem), co-trimoxazole (a sulfonamide), and doxycycline (a tetracycline), to determine the *in vitro* activity against *B. pseudomallei*. Synergy was demonstrated for finafloxacin when used in combination with meropenem at pH 7 in a checkerboard assay and when combined with doxycycline *in vitro* and *in vivo*.

Combining finafloxacin with doxycycline resulted in synergistic activity *in vitro* when compared to the antibiotics used individually. In contrast, combining finafloxacin with co-trimoxazole resulted in antagonistic activity. A study by Ocampo et al. (2014) evaluated combinations of 21 antibiotics to investigate the effect on *E. coli* strain K-12. This was performed in a minimal medium supplemented with 0.2% glucose and 0.1% casamino acids at 30°C. The most relevant combinations to this work are the combinations of ciprofloxacin (a bactericidal antibiotic) and doxycycline (a bacteriostatic antibiotic), ciprofloxacin and trimethoprim (a bacteriostatic antibiotic), and ciprofloxacin and sulfamethoxazole (a bacteriostatic antibiotic), which were shown to be antagonistic, synergistic, and indifferent, respectively (Ocampo et al., 2014). These data conflict with the data generated in this study; however, finafloxacin is known to have superior activity to the second-generation fluoroquinolone ciprofloxacin *in vitro*, *in vivo*, and in clinical trials, the activity which is potentiated at an acidic pH. In addition, *B. pseudomallei* is a very different bacterium to *E. coli*. Interestingly, the components of co-trimoxazole (trimethoprim and sulfamethoxazole) that were evaluated appeared to result in a more positive effect with ciprofloxacin than with doxycycline; however, the components were not evaluated together as co-trimoxazole (Ocampo et al., 2014). Another study showed that a combination of tetracycline and ciprofloxacin was synergistic and bacteriostatic for *Klebsiella pneumoniae* and Enterobacter (Sulieman, 2008).

It is clear that many variables can affect the data generated, including the antibiotic combination, the conditions of the assay, the organism under investigation, and the mechanism of activity of the individual antibiotics. As the finafloxacin-doxycycline combination was shown to be synergistic in time-kill assays in our study, it was down-selected for further characterization *in vivo*.

Doxycycline is a bacteriostatic antibiotic; however, data generated at Dstl and by others have shown that it is active against *B. pseudomallei in vitro* at pH 7 (Thibault et al., 2004; Ross et al., 2018). It has also historically been used to treat patients infected with *B. pseudomallei*. Until 1985, it was used in combination with chloramphenicol and co-trimoxazole for the acute phase of melioidosis (Dance, 2014). Currently, doxycycline is recommended as a second-line agent for the eradication phase of the disease, if co-trimoxazole or co-amoxiclav are not tolerated, which is observed with high doses (Cheng et al., 2008; Sullivan et al., 2019).

To improve the level of protection offered with finafloxacin against *B. pseudomallei* when administered at 24 h post-infection (Barnes et al., 2017), orally delivered doxycycline was co-administered with finafloxacin, and the window of opportunity was increased to 36 h post-infection. The data presented above suggest that there is no difference in the level of protection offered when finafloxacin is combined with doxycycline when compared to finafloxacin or doxycycline monotherapy when initiated at 24 h post-infection. Minimal clinical scores were observed within these animals throughout this study, with limited weight loss post-treatment cessation. More number of mice treated with the combination were clear of detectable bacteria at the end of the study period compared with the finafloxacin and doxycycline monotherapies (7/8, 4/9, and 5/8, respectively).

Interestingly, however, when treatment was initiated at 24 h post-infection, there is evidence for improved bacterial control in the animals treated with the combination or finafloxacin when compared to the doxycycline-treated animals. This may be due to the inability of doxycycline to kill the residual bacteria in the absence of a bactericidal antibiotic.

More differences were observed in animals when treatment was initiated at 36 h post-infection. All surviving mice treated with doxycycline had clinical scores at the end of the study, with 56 and 75% of those treated with finafloxacin and the combination not showing signs at the end of the study. In addition, 50% of the surviving mice treated with the combination had no detectable bacteria in their organs. When treatment was delayed, doxycycline was unable to prevent the development of clinical signs once treatment had ceased, probably due to the level of bacteria present in tissues before the treatment was initiated. It is also likely that mice relapse sooner if treated with this antibiotic as it is bacteriostatic in activity, which aligns with all mice having detectable bacteria in the organs at the end of the study. There was also a benefit for treating with the combination compared to finafloxacin alone. Finafloxacin was more efficacious when compared to doxycycline.

There are other reasons why doxycycline may not be as effective against *B. pseudomallei* in this study. It is possible that doxycycline is not as active in infected body sites that are more acidic in nature. This was shown to be the case *in vitro* with *C. burnetii*, and the activity was improved at a higher pH (Smith et al., 2019). Another variable that could be investigated further is the dose of doxycycline that needs to be administered. The dose used in this study (100 mg/kg, delivered three times daily) was based on the PK profile in Balb/c mice and the potential of toxicity in rodents. The dose chosen was at the lower end of the AUC in humans to reduce the risk of toxicity; however, a further study could investigate a higher dose which would result in a greater AUC. However, other groups that have investigated the use of doxycycline against *B. pseudomallei* infections in mice have used 40–50 mg/kg delivered 1–2 times a day, which is considerably reduced when compared to the dose used in this study (Sivalingam et al., 2008; Thomas et al., 2012; Gelhaus et al., 2013).

This study has demonstrated the positive impact of combining finafloxacin and doxycycline on both survival and control of the bacterial load in tissues. Further studies are warranted to further understand the mechanisms by which these antibiotics work synergistically. In addition, investigating the effect of further increasing the window of opportunity may allow for further differences between the different treatments to be observed.

## Data availability statement

The original contributions presented in this study are included in the article, further inquiries can be directed to the corresponding author.

## Ethics statement

The animal study was reviewed and approved by DSTL Animal Welfare and Ethical Review Body and an approved ACURO appendix.

## Author contributions

KB, CB, TL, SA, MN, and SH conceived the concept and designed the experiments detailed in the manuscript. KB, GB, and MR conducted the experiments. KB, TM, SA, and TL performed the data analysis. KB, TL, TM, and SH wrote the manuscript. All authors reviewed the draft and approved the manuscript for publication.

## References

[B1] Agwuh K. N. MacGowan A. (2006). Pharmacokinetics and pharmacodynamics of the tetracyclines including glycylcyclines. *J. Antimicrob. Chemother.* 58 256–265. 16816396 10.1093/jac/dkl224

[B2] Akova M. Gür D. Livermore D. M. Kocagöz T. Akalin H. E. (1999). *In vitro* activities of antibiotics alone and in combination against *Brucella melitensis* at neutral and acidic pHs. *Antimicrob. Agents Chemother.* 43 1298–1300. 10.1128/AAC.43.5.1298 10223958 PMC89265

[B3] Al-Hasan M. N. Wilson J. W. Lahr B. D. Thomsen K. M. Eckel-Passow J. E. Vetter E. A. (2009). β-Lactam and fluoroquinolone combination antibiotic therapy for bacteremia caused by gram-negative *Bacilli*. *Antimicrob. Agents Chemother.* 53 1386–1394. 10.1128/AAC.01231-08 19164144 PMC2663102

[B4] Barnes K. B. Hamblin K. H. Richards M. I. Laws T. R. Vente A. Atkins H. S. (2017). Demonstrating the protective efficacy of the novel fluoroquinolone finafloxacin against an inhalational exposure to *Burkholderia pseudomallei*. *Antimicrob. Agents Chemother.* 61:7. 10.1128/AAC.00082-17 28438936 PMC5487660

[B5] Barnes K. B. Zumbrun S. D. Halasohoris S. A. Desai P. D. Miller L. L. Richards M. I. (2019a). Demonstration of the broad spectrum *in vitro* activity of finafloxacin against pathogens of biodefence interest. *Antimicrob. Agents Chemother.* 63:12. [Epub ahead of print]. 10.1128/AAC.01470-19 31570393 PMC6879258

[B6] Barnes K. B. Hamblin K. A. Richards M. I. Laws T. R. Vente A. Atkins H. S. (2019b). The fluoroquinolone finafloxacin protects BALB/c mice against an intranasal infection with *Francisella tularensis* strain SchuS4. *Front. Microbiol.* 10:904. 10.3389/fmicb.2019.00904 31118924 PMC6504792

[B7] Barnes K. B. Richards M. I. Laws T. R. Nunez A. Thwaite J. E. Bentley C. (2021). Finafloxacin is an effective treatment for inhalational tularemia and plague in mouse models of infection. *Antimicrob. Agents Chemother.* 65:e02294-20. 10.1128/AAC.02294-20 33753342 PMC8315961

[B8] Chalhoub H. Harding S. V. Tulkens P. M. Van Bambeke F. (2019). Influence of pH on the activity of finafloxacin against extracellular and intracellular *Burkholderia thailandensis, Yersinia pseudotuberculosis and Francisella philomiragia* and on its cellular pharmacokinetics in THP-1 monocytes. *Clin. Microbiol.Inf.* [Epub ahead of print]. 10.1016/j.cmi.2019.07.028 31404671

[B9] Chan T. Y. (1997). Co-trimoxazole-induced severe haemolysis: the experience of a large general hospital in Hong Kong. *Pharmacoepidemiol. Drug Saf.* 6 89–92. 10.1002/(SICI)1099-1557(199703)6:2<89::AID-PDS261>3.0.CO;2-8 15073793

[B10] Cheng A. C. Chierakul W. Chaowagul W. Chetchotisakd P. Limmathurotsakul D. Dance D. A. (2008). Consensus guidelines for dosing of amoxicillin-clavulanate in melioidosis. *Am. J. Trop. Med. Hyg.* 78 208–209. 18256414 PMC3034162

[B11] Collett D. (2015). *Modelling Survival Data in Medical Research.* New York, NY: CRC press.

[B12] Craig W. (1998). Pharmacokinetic/pharmacodynamic parameters: rationale for antibacterial dosing of mice and men. *Clin. Infect. Dis.* 26 1–12. 10.1086/516284 9455502

[B13] Dance D. A. (2014). Treatment and prophylaxis of melioidosis. *Intl. J. Antimicrob. Agents* 43 310–318. 10.1016/j.ijantimicag.2014.01.005 PMC4236584 24613038

[B14] De Backer D. (2003). Lactic acidosis. *Minerva Anestesiol.* 69 281–284. 12766720

[B15] Durukan D. Doyle M. Murray G. Bodiyabadu K. Vodstrcil L. Chow E. P. F. (2020). Doxycycline and sitafloxacin combination therapy for treating highly resistant *Mycoplasma* genitalium. *Emerg. Infect. Dis.* 26 1870–1874. 10.3201/eid2608.191806 32687029 PMC7392426

[B16] Garcia L. S. (2007). “Time kill assay for determining synergy,” in *Clinical Microbiology Procedures Handbook*, 2nd Edn. Washington, DC: American Society for Microbiology Press.

[B17] Gelhaus H. C. Anderson M. S. Fisher D. A. Flavin M. T. Xu Z. Q. Sanford D. C. (2013). Efficacy of post exposure administration of doxycycline in a murine model of inhalational melioidosis. *Sci. Rep.* 3:1146. 10.1038/srep01146 23359492 PMC3556592

[B18] Goh C. Y. Ng S. B. Everett M. Vente A. (2011). “Investigation of *In vitro* antagonistic and synergistic effects of finafloxacin in combination with other antibiotics,” in *Poster Presented at the 51st ICACC*, (Chicago).

[B19] Guyton A. C. (1947). Measurement of the respiratory volumes of laboratory animals. *The Am. J. Physiol.* 150 70–77. 20252828 10.1152/ajplegacy.1947.150.1.70

[B20] Harper G. J. Morton J. D. (1962). A method for measuring the retained dose in experiments on airborne infection. *J. Hyg.* 60 249–257. 13904776 10.1017/s0022172400039504 PMC2134408

[B21] Hartley M. G. Norville I. Richards M. Barnes K. Bewley K. Vipond J. (2021). Finafloxacin, a novel fluoroquinolone, reduces the clinical signs of infection and pathology in a mouse model of Q fever. *Front. Microbiol.* 12:760698. 10.3389/fmicb.2021.760698 34917048 PMC8670379

[B22] Higgins PG Stubbings W Wisplinghoff H Seifert H. (2010). Activity of the investigational fluoroquinolone finafloxacin against ciprofloxacin sensitive and resistant *Acinetobacter baumannii* isolates. Antimicrob. Agents Chemother. 54:1613-1615. 10.1128/AAC.01637-09 20100879 PMC2849356

[B23] Ho J. M. Juurlink D. N. (2011). Considerations when prescribing trimethoprim-sulfamethoxazole. *CMAJ* 183 1851–1858. 21989472 10.1503/cmaj.111152 PMC3216436

[B24] Khosravi Y. Vellasamy K. M. Mariappan V. Ng S.-L. Vadivelu J. (2014). Antimicrobial susceptibility and genetic characterisation of *Burkholderia pseudomallei* isolated from Malaysian patients. *Sci. World J.* 2014:132971. 10.1155/2014/132971 25379514 PMC4213392

[B25] Lemaire S. Van Bambeke F. Tulkens P. (2011). Activity of finafloxacin, a novel fluoroquinolone with increased activity at acid pH, towards extracellular and intracellular *Staphylococcus aureus, Listeria monocytogenes and Legionella pneumophila*. *Int. J. Antimicrob. Agents* 38 52–59. 10.1016/j.ijantimicag.2011.03.002 21596526

[B26] Limmathurotsakul D. Chaowagul W. Chierakul W. Stepniewska K. Maharjan B. Wuthiekanun V. (2006). Risk factors for recurrent melioidosis in northeast Thailand. *Clin. Infect. Dis.* 43 979–986. 16983608 10.1086/507632

[B27] Limmathurotsakul D. Golding N. Dance D. A. Messina J. P. Pigott D. M. Moyes C. L. (2016). Predicted global distribution of *Burkholderia pseudomallei* and burden of melioidosis. *Nat. Microbiol.* 1:15008. 10.1038/nmicrobiol.2015.8 27571754

[B28] Madden D. E. Webb J. R. Steinig E. J. Currie B. J. Price E. P. Sarovich D. S. (2021). Taking the next gen step: Comprehensive antimicrobial resistance detection from *Burkholderia pseudomallei*. *Ebio Medi.* 63:103152. 10.1016/j.ebiom.2020.103152 33285499 PMC7724162

[B29] Ng A. W. Bidani A. Heming T. A. (2004). Innate host defense of the lung: effects of lung-lining fluid pH. *Lung* 182 297–317. 10.1007/s00408-004-2511-6 15742242

[B30] O’Brien D. P. McDonald A. Callan P. Robson M. Friedman N. D. Hughes A. (2012). Successful outcomes with oral fluoroquinolones combined with rifampicin in the treatment of *Mycobacterium ulcerans*: an observational cohort study. *PLoS Negl. Trop. Dis.* 6:e1473. 10.1371/journal.pntd.0001473 22272368 PMC3260310

[B31] Ocampo P. S. Lázár V. Papp B. Arnoldini M. (2014). Abel zur Wiesch P, Busa-Fekete R, Fekete G, Pál C, Ackermann M, Bonhoeffer S. Antagonism between bacteriostatic and bactericidal antibiotics is prevalent. *Antimicrob. Agents Chemother.* 58 4573–4582. 10.1128/AAC.02463-14 24867991 PMC4135978

[B32] Patel H. Andresen A. Vente A. Heilmann H. D. Stubbings W. Seiberling M. (2011). Human pharmacokinetics and safety profile of finafloxacin, a new fluoroquinolone antibiotic, in healthy volunteers. *Antimicrob. Agents Chemother.* 55 4386–4393. 10.1128/AAC.00832-10 21709093 PMC3165282

[B33] Peyrusson F. Whelan A. O. Hartley M. G. Norville I. H. Harding S. V. Van Bambeke F. (2021). Intracellular activity of antibiotics against *Coxiella burnetii* in a model of activated human THP-1 cells. *Antimicrob. Agents Chemother.* 65:e0106121. 10.1128/AAC.01061-21 34543094 PMC8597764

[B34] Punnia-Moorthy A. (1987). Evaluation of pH changes in inflammation of the subcutaneous air pouch lining in the rat, induced by carrageenan, dextran and *Staphylococcus aureus*. *J. Oral Pathol.* 16 36–44. 10.1111/j.1600-0714.1987.tb00674.x 2435877

[B35] Rao C. Zhiqiang H. Jiangao C. Tang M. Chen H. Lu X. (2019). Molecular epidemiology and antibiotic resistance of *Burkholderia pseudomallei* isolates from Hainan. *China Med.* 98:e14461. 10.1097/MD.0000000000014461 PMC6831382 30817562

[B36] Ross B. N. Myers J. N. Muruato L. A. Tapia D. Torres A. G. (2018). Evaluating new compounds to treat *Burkholderia pseudomallei* infections. *Front. Cell. Infect. Microbiol.* 8:210. 10.3389/fcimb.2018.00210 30013953 PMC6036294

[B37] Schnetterle M. Gorgé O. Nolent F. Boughammoura A. Sarilar V. Vigier C. (2021). Genomic and RT-qPCR analysis of trimethoprim-sulfamethoxazole and meropenem resistance in *Burkholderia pseudomallei* clinical isolates. *PLoS Neg. Trop. Dis.* 15:e0008913. 10.1371/journal.pntd.0008913 PMC7909661 33592059

[B38] Smith R. P. Baltch A. L. Hammer M. C. Conroy J. V. (1988). *In vitro* activities of PD 117,596 and reference antibiotics against 448 clinical bacterial strains. *Antimicrob. Agents Chemother.* 32 1450–1455. 10.1128/AAC.32.9.1450 3196008 PMC175890

[B39] Stubbings W. Leow P. Chee Yong G. Goh F. Korber-Irrgang B. Kreksken M. (2011). *In vitro* spectrum of activity of finafloxacin, a novel, pH-activated fluoroquinolone, under standard and acidic conditions. *Antimicrob. Agents Chemother.* 55 4394–4397. 10.1128/AAC.00833-10 21709094 PMC3165343

[B40] Sulieman S. M. (2008). The effect of using combinations of commonly used antibiotics in Sudan on resistant gram negative bacterial isolates. *Intl. J. Infect. Dis.* 12:E123.

[B41] Sullivan R. P. Ward L. Currie B. J. (2019). Oral eradication therapy for melioidosis; important but not without risks. *Intl. J. Infect. Dis.* 80 111–114. 10.1016/j.ijid.2019.01.019 30659921

[B42] Sullivan R. P. Marshall C. S. Anstey N. M. Ward L. Currie B. J. (2020). Review and revision of the 2015 darwin melioidosis treatment guideline; paradigm drift not shift. *PLoS Negl. Trop. Dis.* 14:e0008659. 10.1371/journal.pntd.0008659 32986699 PMC7544138

[B43] Sivalingam S. P. Sim S. H. Jasper L. C. W. Wang D. Liu Y. Ooi E. E. (2008). Pre- and post-exposure prophylaxis of experimental *Burkholderia pseudomallei* infection with doxycycline, amoxicillin/clavulanic acid and co-trimoxazole. *J. Antimicrob. Chemother.* 61 674–678. 10.1093/jac/dkm527 18192684

[B44] Smith C. B. Evavold C. Kersh G. J. (2019). The effect of ph on antibiotic efficacy against *Coxiella burnetii* in axenic media. *Sci. Rep.* 9:18132. 10.1038/s41598-019-54556-6 31792307 PMC6889355

[B45] Thibault F. M. Hernandez E. Vidal D. R. Girardet M. Cavallo J.-D. (2004). Antibiotic susceptibility of 65 isolates of *Burkholderia pseudomallei* and *Burkholderia mallei* to 35 antimicrobial agents. *J. Antimicrob. Chemother.* 54 1134–1138. 10.1093/jac/dkh471 15509614

[B46] Thomas R. J. Davies C. Nunez A. Hibbs S. Eastaugh L. Harding S. (2012). Particle-size dependent effects in the Balb/c murine model of inhalational melioidosis. *Front. Cell. Infect. Microbiol.* 2:101. 10.3389/fcimb.2012.00101 22919690 PMC3417579

[B47] Vente A. Bentley C. Lückermann M. Tambyah P. Dalhoff A. (2018). Early clinical assessment of the antimicrobial activity of finafloxacin compared to ciprofloxacin in subsets of microbiologically characterized isolates. *Antimicrob. Agents Chemother.* 62 e2325–e2317. 10.1128/AAC.02325-17 29339393 PMC5913966

[B48] Wagenlehner F. Nowicki M. Bentley C. Lückermann M. Wohlert S. Fischer C. (2018). Explorative randomized phase II clinical study of the efficacy and safety of finafloxacin versus ciprofloxacin for treatment of complicated urinary tract infections. *Antimicrob. Agents Chemother.* 62 e2317–e2317. 10.1128/AAC.02317-17 29339395 PMC5913919

[B49] Wayne P. (1999). Methods for determining bactericidal activity of antimicrobial agents: approved guideline M-26-A. *Clin. Lab. Stand. Inst.* 19:18. 10.1128/JCM.37.6.1881-1884.1999 10325341 PMC84976

[B50] Yang L. Wang K. Li H. Denstedt J. D. Cadieux P. A. (2014). The influence of urinary pH on antibiotic efficacy against bacterial uropathogens. *Urology* 84 .e1–.e7. 25168568 10.1016/j.urology.2014.04.048

